# The course and determinants of post-traumatic stress over 12 months after hospitalization for COVID-19

**DOI:** 10.3389/fpsyt.2022.931349

**Published:** 2022-07-15

**Authors:** Knut Stavem, Trond Heir, Toril Dammen, Eivind Brønstad, Tøri Vigeland Lerum, Michael T. Durheim, Kristine M. A. Lund, Bernt B. Aarli, Gunnar Einvik

**Affiliations:** ^1^Department of Pulmonary Medicine, Akershus University Hospital, Lørenskog, Norway; ^2^Institute of Clinical Medicine, University of Oslo, Oslo, Norway; ^3^Health Services Research Unit, Akershus University Hospital, Lørenskog, Norway; ^4^Norwegian Centre for Violence and Traumatic Stress Studies, Oslo, Norway; ^5^Division of Mental Health and Addiction, Oslo University Hospital, Oslo, Norway; ^6^Thoracic Department, St. Olavs Hospital, Trondheim, Norway; ^7^Department of Circulation and Medical Imaging, Faculty of Medicine and Health Sciences, Norwegian University of Science and Technology (NTNU), Trondheim, Norway; ^8^Department of Pulmonary Medicine, Oslo University Hospital Ullevål, Oslo, Norway; ^9^Department of Respiratory Medicine, Oslo University Hospital Rikshospitalet, Oslo, Norway; ^10^Department of Infectious Diseases, Østfold Hospital Trust Kalnes, Grålum, Norway; ^11^Department of Thoracic Medicine, Haukeland University Hospital, Bergen, Norway; ^12^Department of Clinical Science, University of Bergen, Bergen, Norway

**Keywords:** post-traumatic stress disorder (PTSD), COVID-19, PCL-5 questionnaire, cohort, follow-up, medium-term

## Abstract

**Objective:**

To assess the trajectory of symptoms and symptom-defined post-traumatic stress disorder (PTSD) from 1.5 to 12 months after hospitalization for COVID-19 and determine risk factors for persistent symptoms and PTSD.

**Methods:**

This was a prospective cohort study of consecutive patients discharged after hospitalization for COVID-19 before 1 June 2020 in six hospitals in Southern Norway. Symptom-defined PTSD was assessed by the post-traumatic stress disorder (PTSD) checklist for DSM-5 (PCL-5) at 1.5, 3 and/or 12 months after hospitalization, using DSM-5 criteria. Changes in PCL-5 symptom score and the prevalence of PTSD were analyzed with multivariable mixed models.

**Results:**

In total, 388 patients were discharged alive, and 251 (65%) participated. Respondents had a mean (SD) age of 58.4 (14.2) years, and 142 (57%) were males. The prevalence of symptom-defined PTSD was 14, 8, and 9% at 1.5, 3, and 12 months, respectively. WHO disease severity for COVID-19 was not associated with PCL-5 scores. Female sex, lower age and non-Norwegian origin were associated with higher PCL-5 scores. The odds ratio (OR) (95%CI) for PTSD was 0.32 (0.12 to 0.83, *p* = 0.019) at 3 months and 0.38 (0.15 to 0.95, *p* = 0.039) at 12 months compared to 1.5 months. There was no association between PTSD and WHO severity rating.

**Conclusions:**

The level of PTSD symptoms decreased from 1.5 to 3 months after hospitalization, but did not decrease further to 12 months, and there was no association between PTSD symptoms and COVID-19 disease severity.

## Introduction

Post-traumatic stress disorder (PTSD) is a chronic and debilitating mental condition that may develop in response to traumatic events that involve a life-threatening component, including acute medical diseases. A meta-analysis reported that 17–44% of critical illness survivors reported clinically important PTSD symptoms, and that symptoms of PTSD were associated with pre-ICU psychopathology, but not with age, disease severity, or ICU length of stay (1).

During the severe acute respiratory syndrome (SARS) outbreak in 2002–2004, many recovered SARS patients showed symptoms of PTSD at 1 and 3 months after discharge from hospital, and 4–5% satisfied the criteria for PTSD (2), although higher rates have been reported (3). Furthermore, the level of hypoxia is associated with PTSD symptoms (2). The prevalence of PTSD 12 months after hospitalization for the Middle East respiratory syndrome (MERS) were even higher, with 24% reporting severe or very severe PTSD (4).

Patients hospitalized for COVID-19 are characterized by fever, cough, dyspnea, chest pain, or confusion, and typically 10–20% of hospitalized patients have critical illness requiring care in the intensive unit (5, 6). Until June 2022, cumulatively 15.8% of patients hospitalized for COVID-19 in Norway required ICU care (7).

Recent studies have reported significant PTSD symptoms during and after hospitalization for COVID-19 among 2–40 % of patients with follow-up of 1–6 months, and just recently 12–15 months, in cross-sectional or cohort studies (8–20). Prevalence rates vary according to time after acute COVID-19, assessment method, sample selection, and geographical region. During follow-up, female sex and continued symptoms, but not age or disease severity, were associated with PTSD (20).

To understand the course of stress reactions, repeated measurements of PTSD symptoms during the convalescence of COVID-19 and longer observation times are necessary (9, 17). This study investigated the course of symptoms of PTSD over 12 months after COVID-19 onset, focusing on the change in symptoms and possible association with disease severity.

## Materials and Methods

### Design and population

This was a multicenter study, inviting patients 2–6 weeks after discharge from six major hospitals in Norway after COVID-19, before 1 June 2020. All patients >18 years of age with a discharge diagnosis (International Statistical Classification of Diseases and Related Health Problems 10) of U07.1, U07.2, or J12.x were eligible. Among all 454 survivors, we excluded 15 patients living outside the hospitals' catchment areas, 13 unable to provide informed consent, and 38 participating in a conflicting COVID-19 treatment trial. In total, 251 (65%) of 388 eligible patients provided data for this study.

The Regional Committee for Medical and Health Research Ethics, Health Region South East (no. 2020/125384) and data protection officers at participating centers approved the study. Written informed consent was obtained by mail or a secure web-form. The study is registered with ClinicalTrials.gov (NCT04535154).

### Data collection

As this was an observational study, clinical treatment was up to each hospital. However, during the study follow-up visits, we used a standard protocol.

Data were collected at four time points: (i) Previous medical history and data during COVID-19 hospitalization were extracted from the electronic medical records (EMR); (ii) about 1.5 month after hospitalization, participants responded to a paper or on-line questionnaire; all participants returned to the respective hospitals' outpatient clinics for a visit, including self-completed questionnaires at (iii) 3 and (iv) 12 months after hospitalization.

### Demographics, clinical variables, and comorbidity

Basic demographics, symptoms, Charlson's comorbidity index (21), and severity of COVID-19 were extracted from the EMR by study physicians/nurses. COVID-19 severity was classified using a WHO ordinal severity score (22): 3 no oxygen supplementation, 4 oxygen supplementation, 5 high-flow oxygen or non-invasive ventilation, or 6–7 mechanical ventilation. We obtained supplementary information on socio-demographics, height, weight, and smoking history by self-report at the 3-month visit.

### Assessment of PTSD

Symptoms of PTSD were assessed using a Norwegian version of the PCL-5 (PTSD Checklist for DSM-5) questionnaire (23). The PCL-5 contains 20 items on an ordinal scale (0 to 4), which are summed to a total score (range 0–80, with 80 denoting maximal symptoms). The PCL-5 also has an alternative scoring algorithm fitted to the five clusters of the DSM-5 PTSD criteria (24). We used the DSM-5 criteria scoring to define symptom-defined PTSD.

### Statistical analyses

We analyzed data from respondents with at least one response to PCL-5 (*n* = 251). Descriptive statistics are presented as mean (SD), median (25th to 75th percentile) or number (%). We present PTSD symptoms as continuous scores on the PCL-5 total scale and the prevalence of PTSD using DSM-5 scoring.

We conducted longitudinal analyses using mixed models, thereby accounting for missing values and enabling us to use all available data. For PCL-5 total scores, we used a linear mixed model with random effect (intercept) at the patient level, and an unstructured covariance structure. The model was fitted using maximum likelihood. Because the distribution of PCL-5 total score was highly skewed with many subjects having a score of 0, we tried log and square-root transformations of the PCL-5 total scores. However, this did not improve the distribution of the residuals or the model fit. Therefore, we used untransformed values as the dependent variable and bootstrapping with 10,000 iterations to estimate 95% confidence intervals and *p*-values. We included independent variables selected *a priori*: occasion (1.5, 3, or 12 months), age (per year), sex (male vs. female), living alone (married/cohabiting vs. single/divorced/widowed), born in Norway (yes vs. no), education (university level vs. lower level), Charlson comorbidity score (0, 1, ≥2), and WHO ordinal severity score (3, 4, 5–7).

Adding a random effect at the hospital level to the model only marginally influenced the coefficients and did not alter the results. The intraclass correlation coefficient (ICC) at the hospital level was only 0.029 compared to 0.657 at the patient level. Therefore, we omitted the hospital level in further analysis. In a supplementary analysis, we repeated the linear mixed models for PCL-5 total scores with the same approach and the same variables.

PTSD was analyzed using a logistic mixed model, with random effect (intercept) at the patient level, and an unstructured covariance structure. Here we only used occasion (1.5, 3, or 12 months) and WHO ordinal severity score as covariates, because of the low number of events.

We used Stata version 17.0 (Stata Corporation, College Station, TX) or R (The R Foundation for Statistical Computing) for all statistical analyses. We chose a 5% significance level.

## Results

The participants had a mean age of 58.4 years, range 16.6 to 91.3 years. Overall, 35/142 men (25%) and 13/109 women (12%) had been admitted to the ICU. Further demographic and clinical characteristics of the participants are shown in Table 1.

**Table 1:** Descriptive statistics for respondents (*N* = 251), number (%) unless otherwise stated.

	***n***	
Age (years), mean (SD)		58.4 (14.2)
Sex, male		142 (57)
Education, university level (>13 years)	236	129 (55)
Norwegian origin	226	163 (72)
Smoking status, current/previous		121 (48)
Living alone (unmarried/divorced/widowed)	226	62 (27)
Body mass index (kg/m2), mean (SD)	235	28.2 (4.6)
**Comorbidity**		
Myocardial infarction		14 (6)
Congestive heart failure		11 (4)
Peripheral vascular disease		4 (2)
Cerebrovascular accident or transitory ischemic attack		6 (2)
Dementia/chronic cognitive deficit		0 (0)
Chronic obstructive pulmonary disease		8 (3)
Connective tissue disease		3 (1)
Peptic ulcer disease		13 (5)
Liver disease, incl cirrhosis		3 (1)
Diabetes		21 (8)
Hemiplegia		0 (0)
Moderate to severe chronic kidney disease, on dialysis, or transplant		7 (3)
Solid tumor		10 (4)
Leukemia		2 (1)
Lymphoma		0 (0)
AIDS		0 (0)
Charlson comorbidity index, weighted, mean (SD)		0.49 (0.99)
**Charlson comorbidity index**		
0		180 (72)
1		41 (16)
≥2		30 (12)
**Status at hospital admission**		
Systolic blood pressure (mmHg), mean (SD)	247	134 (18.4)
Diastolic blood pressure (mmHg), mean (SD)	247	78.3 (11.4)
Body temperature (°C), mean (SD)	244	37.6 (1.1)
Pulse rate (per min), mean (SD)	245	85.6 (19.2)
Respiratory rate (per min), mean (SD)	240	23.9 (8)
**Arterial blood gas at admission**		
pH, mean (SD)	220	7.46 (0.05)
P_a_O_2_ (kPa), mean (SD)	222	9.9 (2.2)
P_a_CO_2_ (kPa), mean (SD)	222	4.4 (0.9)
**WHO severity rating**		
3 Hospitalized, no oxygen therapy		87 (35)
4 Oxygen by mask or nasal prongs		121 (48)
5 Non-invasive ventilation or high-flow oxygen		10 (4)
6 Intubation and mechanical ventilation		32 (13)
7 Ventilation + ECMO		1 (0)
NEWS2 score at admission	234	3.9 (2.6)
Admission to intensive care unit		48 (19)
**Times and length of stay (days), median (25th to 75th percentile)**		
Hospital length of stay	249	6 (3 to 11)
Length of stay in intensive care unit	46	10.5 (5 to 15)
**Time from hospital admission to response**		
Survey 1 (1.5 months)*	192	46 (29.5 to 56.5)
Survey 2 (3 months)*	173	97 (84 to 119)
Survey 3 (12 months)*	168	386 (358.5 to 406)

* *Only for respondents in the respective surveys*.

*ECMO, extracorporeal membrane oxygenation*.

**Figure 1:**
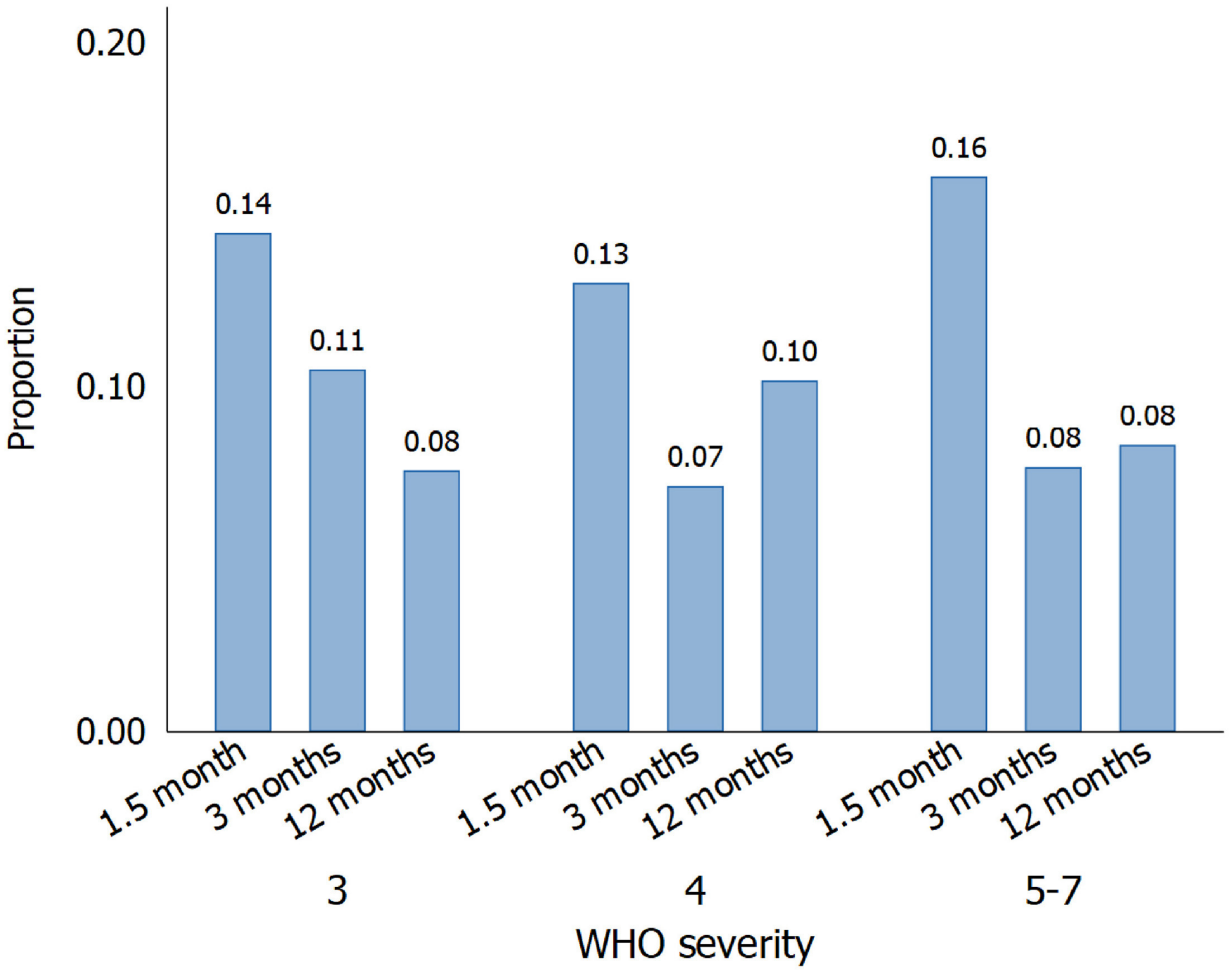
Proportions of hospitalized COVID-19 patients with symptom-defined PTSD (DSM-5 scoring) over time, according to WHO disease severity: 3, no oxygen requirement (*n* = 87); 4, oxygen by mask or nasal prongs (*n* = 121); 5–7, high-flow oxygen, non-invasive ventilation or intubation/mechanical ventilation (*n* = 43).

The mean (SD) PCL-5 total scores were 14.2 (14.2), 11.2 (12.9), and 10.4 (12.4) at 1.5, 3, and 12 months, respectively. Symptom-defined PTSD was present in 27 (14%), 18 (8%), and 19 (9%) of the patients at the same time points, but there was no association with disease severity (Figure 1).

**Figure 2:**
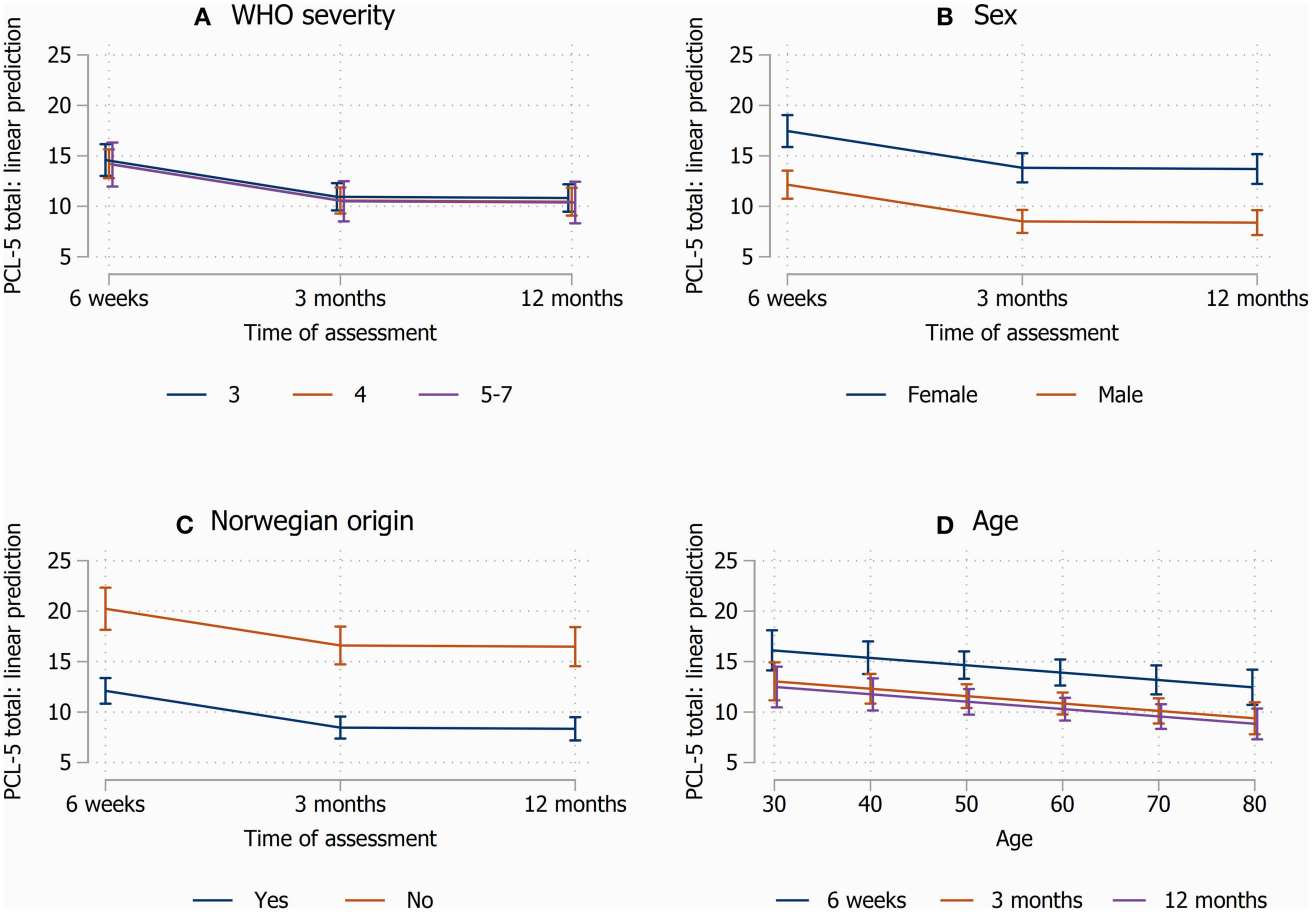
Margin plots from the linear mixed models, illutrating the effects of covariates on estimated PCL-5 scores over time. **(A)** WHO ordinal disease severity, **(B)** sex, **(C)** Norwegian origin, and **(D)** age. Other covariates than those shown were kept at mean values.

In multivariable analysis of continuous PCL-5 scores, 3-month and 12-month scores were lower than 1.5-month scores, but there was no association with COVID-19 severity (Table 2, Figure 2). However, female gender, non-Norwegian origin, and lower age were associated with increased PCL-5 total scores.

**Table 2:** Predictors of PCL-5 total symptom score, multivariable linear mixed model (*n* =565 observations, 225 patients).

**Fixed effects**	**Coef**.**	**95% Conf. interval**	***P***
**Occasion**			
1.5 months*	0		
3 months	−3.64	(−5.30 to −1.98)	<0.001
12 months	−3.76	(−5.51 to −2.01)	<0.001
**WHO severity rating**			
3* No oxygen use	0		
4 Oxygen use	0.82	(−0.68 to 2.32)	0.29
5–7 High flow, ventilatory support	1.06	(−1.15 to 3.27)	0.35
**Sex**			
Female*	0		
Male	−5.90	(−7.49 to −4.31)	<0.001
Age, per year	−0.07	(−0.13 to −0.02)	0.008
**Education**			
Lower level*	0		
University level	1.17	(−0.37 to 2.71)	0.138
**Marital status**			
Married/cohabiting*	0		
Single/divorced/widowed	1.14	(−0.50 to 2.77)	0.174
**Norwegian origin?**			
Yes*	0		
No	7.83	(5.79 to 9.88)	<0.001
**Charlson comorbidity index**			
0*	0		
1	2.76	(0.74 to 4.78)	0.007
≥2	0.15	(−2.04 to 2.35)	0.89
**Random effects**	SD		
Participant (intercept)	10.03		
Residual	6.78		
**Model statistics**			
Intraclass correlation coefficient	0.686		
Akaike's information criterion	4306.8		
Marginal/conditional *R*^2^	0.149/0.733		

* *Baseline category*.

** *Unstandardized beta coefficient*.

Stratified analysis according to sex, showed a similar development in PTSD symptoms over time for men and women, and more PTSD symptoms with non-Norwegian origin (Supplementary Table 1). However, the results for educational level seemed inconsistent, with more symptoms with higher education among women, but less symptoms with higher education among men. Being single/divorced was associated with more symptoms only among men. Finally, the association for Charlson comorbidity index was different for men and women, with score ≥2 being associated with less PTSD symptoms only among men.

The multivariable logistic model (*n* = 618 observations, 221 patients) showed odds ratios (OR) (95%CI) for PTSD of 0.32 (0.12 to 0.83, *p* = 0.019) at 3 months and 0.38 (0.15 to 0.95, *p* = 0.039) at 12 months compared to 1.5 months, and 0.93 (0.26 to 3.29) for WHO severity rating 4 and 1.01 (0.19 to 5.38) for severity 5–7 compared to severity 3, respectively. ICC was 0.745.

## Discussion

This study has shown a prevalence of PTSD of 14% at 1.5 months, declining to 8–9% after 3–12 months. Furthermore, there was no association between the severity of acute COVID-19 and PTSD symptoms or prevalence at follow-up.

The prevalence of PTSD in this study was in line with studies with similar populations 2–12 months after hospitalization for COVID-19 (10, 11, 13–17), but lower than in some populations with more severe disease (8, 12, 18, 25). However, rates of PTSD disorders following COVID-19 as low as 3.8% has been reported in combined hospitalized/non-hospitalized samples (26).

The differences in prevalence of PTSD between our study and some other studies may possibly be explained by the use of different assessment methods, or populations.

The present study recruited a large proportion of all hospitalized patients in strict geographical catchment areas and may therefore be less affected by selection bias, e.g., healthy non-participant bias in studies recruiting based on lasting symptoms. This, and some other studies (11, 13, 15–17), may therefore be more representative of all hospitalized patients.

Symptoms of PTSD improved from 1.5 to 3 months, in line with reports from some studies from 1 to 2, or 1 to 3 months (17, 27–29), but not in others (30). There is less information on change in PTSD symptoms over as long as 12 months. One study reported the presence of moderate PTSD symptoms in 12% and severe in 6.5% 12 months after hospitalization, and these rates were unchanged from 4 months after hospitalization (16), as from 3 to 12 months in the present study. In contrast, studies have reported an increase in PTSD symptoms scores from 3 to 6 months after hospitalization (31), or from baseline to follow-up after 24–60 weeks in subjects recruited from advertisements or referrals in the community (32). Recently Mazza et al. reported a reduction in PTSD symptoms over time from 1 month to 12 months, although the change from 6 to 12 months seemed to be small (18).

In the present study we found no significant association between the severity of COVID-19 during hospitalization, i.e., graded requirement for oxygen treatment, and the total score of PCL-5 during the follow-up. This, however, supports previous reports of no association between disease severity and adverse mental outcomes in COVID-19 (20).

In contrast, female sex was associated with higher symptom score, in line with findings in other studies (20) and PTSD in general (33). It is possible that young people are less prepared for being affected by acute severe illness (34), and that birth place outside Norway may be related to higher probability of previous PTSD, lower confidence in authorities or information given, or language difficulties.

A Charlson comorbidity index of 1, but not of ≥ 2, was associated with PCL-5 scores, which was most marked in the analysis in males. We do not have a good explanation for this. It could be by chance, content of the medical records, or coding issues. Some of the comorbidities might also be more important in this context, than what is reflected in the weights in this index. In the stratified analysis, this inconsistency was present only among men, who constituted 57% of the sample, and an even larger percentage of those with severe disease requiring ICU admission. In the stratified analyses, the statistical power was smaller, in particular among women, which limits conclusions to be made.

Strengths of the study are the prospective design with multiple assessments, inclusion of several large centers covering approximately 50% of the Norwegian population, and the long follow-up. The use of questionnaires and symptom-defined PTSD, instead of diagnostic interviews for assessment of PTSD represents a limitation, as in most other studies. The response rate among participants was not optimal, and may have caused a bias, and the number of patients with severe disease was small, reducing the statistical power. We did not register whether PTSD was present prior to COVID-19. However, the PCL-5 items were specifically linked to the infectious disease.

In this study the prevalence of PTSD was low. There was an early improvement in symptoms of PTSD, but no further improvement beyond 3 months. In general, about half of PTSD cases remit within 6 months, and the probability of remission does not vary much across exposure types (35). A similar remission rate of PTSD symptoms over a much shorter time period in our study suggests that COVID-19 PTSD may have a better prognosis than PTSD from most other causes.

In conclusion, the symptom load of PTSD declined from 1.5 to 3 months, with no further decline from 3 to 12 months. Symptoms of PTSD were not associated with COVID-19 severity.

## Data Availability Statement

The raw data supporting the conclusions of this article will be made available by the authors, without undue reservation.

## Ethics Statement

The studies involving human participants were reviewed and approved by Regional Committee for Medical and Health Research Ethics, Health Region South East. The patients/participants provided their written informed consent to participate in this study.

## Author Contributions

GE and KS conceived and designed the study, with assistance from TD and TH. EB, TL, MD, KL, and BA contributed to data collection. KS, GE, and TH conducted data analysis. KS, GE, TH, and TD contributed to interpretation of the data. KS, GE, TD, TH, EB, TL, MD, KL, and BA critically reviewed and commented on the paper. All authors approved the final version.

## Conflict of interest

The authors declare that the research was conducted in the absence of any commercial or financial relationships that could be construed as a potential conflict of interest.

## Publisher's Note

All claims expressed in this article are solely those of the authors and do not necessarily represent those of their affiliated organizations, or those of the publisher, the editors and the reviewers. Any product that may be evaluated in this article, or claim that may be made by its manufacturer, is not guaranteed or endorsed by the publisher.
