# Supplementary material for: The course and determinants of post-traumatic stress over 12 months after hospitalization for COVID-19
Source: Front Psychiatry. 2022 Jul 15;13:931349. doi: 10.3389/fpsyt.2022.931349 (PMC9334651; doi:10.3389/fpsyt.2022.931349)
Supplement: Supplementary file 1 [file Data_Sheet_1.PDF]

Supplementary Table 1. Predictors of PCL-5 total symptom score, multivariable linear mixed models in strata according to sex.

	Women (98 patients, 233 observations)			Men (127 patients, 332 observations)		
Fixed effects	Coef.**	95% Conf. Interval	*P*	Coef.**	95% Conf. Interval	*P*
Occasion						
1.5 month*	0			0		
3 months	-3.52	(-6.13 to -0.90)	0.008	-3.80	(-5.93 to -1.67)	<0.001
12 months	-4.06	(-6.52 to -1.61)	0.001	-3.60	(-5.94 to -1.25)	0.003
Who severity rating						
3* No oxygen use	0			0		
4 Oxygen use	2.44	(-0.02 to 4.90)	0.052	-1.11	(-2.97 to 0.76)	0.25
5–7 High flow, ventilatory support	4.98	(-1.24 to 11.19)	0.116	-0.50	(-2.77 to 1.77)	0.67
Age, per year	-0.02	(-0.10 to 0.07)	0.73	-0.06	(-0.13 to 0.01)	0.073
Education						
Lower level*	0			0		
University level	6.04	(2.56 to 9.52)	0.001	-1.93	(-3.83 to -0.02)	0.047
Marital status						
Married/cohabiting*	0			0		
Single/divorced/widowed	-0.31	(-2.83 to 2.21)	0.81	2.56	(0.20 to 4.92)	0.034
Norwegian origin?						
Yes*	0			0		
No	8.92	(5.55 to 12.29)	<0.001	7.01	(4.44 to 9.57)	<0.001
Charlson com.index (0,1,2+)						
0*	0			0		
1	2.44	(-2.38 to 7.26)	0.32	3.64	(1.40 to 5.89)	0.001
≥2	3.27	(-0.76 to 7.31)	0.112	-2.38	(-4.41 to -0.35)	0.022
Random effects	SD			SD		
Participant (intercept)	11.57			8.18		
Residual	6.60			6.88		
Model statistics						
Intraclass correlation coefficient	0.754			0.585		
Akaike's information criterion	1769.1			2442.5		
Marginal/conditional R^2	0.124/0.785			0.175/0.658		

* Baseline category

** Unstandardized beta coefficient
